# Research progress on the mechanisms and clinical applications of neuromodulation techniques for intervening in interhemispheric imbalance after stroke

**DOI:** 10.3389/fneur.2026.1890806

**Published:** 2026-09-07

**Authors:** Yu Cao, Zhiheng Zhu, Weiwei Guo, Yumin Zhou, Xia Bi

**Affiliations:** Department of Rehabilitation Medicine, Shanghai University of Medicine and Health Sciences Affiliated Zhoupu Hospital, Shanghai, China

**Keywords:** interhemispheric inhibition, motor recovery, neuromodulation, neurorehabilitation, optogenetics, stroke, transcranial magnetic stimulation

## Abstract

Post-stroke interhemispheric inhibitory imbalance acts as one of the major contributing pathological factors driving motor impairment, rather than a universal primary pathological mechanism across all stroke subtypes. This narrative review systematically summarizes preclinical and clinical neuromodulation interventions including optogenetic manipulation and transcranial magnetic stimulation, grounded in the interhemispheric inhibition (IHI) theory and bimodal balance–recovery framework. We elaborate how these techniques restore excitatory/inhibitory (E/I) homeostasis via regulating neurotransmitter concentrations, modifying receptor expression, and modulating targeted transcallosal neuronal circuits, while explicitly acknowledging inconsistent research findings across measurement modalities, stroke recovery stages and patient subgroups. We further integrate quantitative clinical trial data, effect sizes and evidence grading to analyze current clinical translation status of repetitive transcranial magnetic stimulation (rTMS), transcranial direct current stimulation (tDCS) and continuous theta-burst stimulation (cTBS). Critical unresolved challenges include translational gaps between cell-specific optogenetic mechanisms and human non-invasive brain stimulation (NIBS), heterogeneous patient responses, and lack of unified quantitative thresholds defining pathological interhemispheric imbalance. Finally, we propose prospective research directions covering multi-network targeted modulation, multimodal predictive biomarkers, combined pharmacology-neuromodulation strategies and large stratified randomized controlled trials to advance precision individualized stroke neurorehabilitation.

## 1 Introduction

Stroke remains the leading cause of permanent disability in adult populations worldwide, and functional recovery largely depends on adaptive neuroplastic reorganization within residual intact neural circuits (1). One key maladaptive neural phenomenon restricting post-stroke functional restoration is disrupted interhemispheric equilibrium, characterized by reduced cortical excitability in the ipsilesional hemisphere and excessive transcallosal inhibitory outflow originating from the contralesional hemisphere toward the injured side (2). This pathological IHI asymmetry is not a secondary epiphenomenon but a major contributor to persistent motor dysfunction, yet its magnitude and clinical impact vary drastically based on lesion topography, corticospinal tract integrity, and time post-stroke.

Multimodal neurophysiological assessments combining transcranial magnetic stimulation and electroencephalography (TMS-EEG) can capture sensitive cortical biomarkers reflecting interhemispheric imbalance. In healthy individuals, reciprocal balanced inhibition exists between bilateral primary motor cortices; after stroke, stimulation of the contralesional hemisphere retains robust inhibitory control over the ipsilesional cortex, while reciprocal inhibition from the injured hemisphere is markedly attenuated or absent, forming stable pathological interhemispheric asymmetry undetected in healthy cohorts (2). Importantly, the severity of IHI asymmetry correlates with motor recovery outcomes: patients with milder interhemispheric imbalance achieve better paretic hand motor strength recovery (2). This dysregulation extends beyond motor networks—interhemispheric perfusion asymmetry in middle cerebral artery territories predicts persistent working memory deficits even years after pediatric stroke (3), confirming broad inhibitory imbalance across sensorimotor and cognitive circuits.

The structural foundation of interhemispheric communication relies on transcallosal fibers within the corpus callosum, mediating both facilitatory and inhibitory signal exchange between homotopic cortical regions to coordinate integrated sensorimotor and cognitive processing. Under physiological conditions, parvalbumin-positive (PV+) interneurons maintain balanced transcallosal inhibitory circuits to sustain bilateral hemispheric coordination (4). Stroke injury disrupts this homeostatic circuit, triggering pathological over-inhibition from the contralesional to ipsilesional hemisphere, quantifiable via TMS metrics including ipsilateral silent period (iSP) (5). Such maladaptive transcallosal suppression constrains neuroplastic potential within peri-infarct tissue. Consistent evidence confirms that favorable motor recovery accompanies restored interhemispheric activity balance, manifested as elevated intrahemispheric coherence within lesioned sensorimotor networks and reduced compensatory hyperactivation in the unaffected hemisphere (1). Therefore, correcting pathological interhemispheric imbalance has become a core therapeutic target for stroke rehabilitation, aiming to reshape cortical excitability and establish a permissive environment for experience-dependent neuroplasticity.

Non-invasive neuromodulation has emerged as a translational tool to directly remodel dysfunctional transcallosal signaling. Core modalities including repetitive transcranial magnetic stimulation (rTMS) and transcranial direct current stimulation (tDCS) enable bidirectional tuning of focal cortical excitability (6). The therapeutic framework of neuromodulation is rooted in the interhemispheric competition hypothesis: functional recovery can be promoted either by suppressing overactive contralesional motor cortex (low-frequency inhibitory rTMS / cTBS) or boosting hypoexcitable ipsilesional cortex (high-frequency excitatory rTMS / anodal tDCS), ultimately rebalancing bilateral cortical excitability (7). For instance, 1 Hz low-frequency rTMS delivered to contralesional primary motor cortex (cM1) reduces its transcallosal inhibitory drive toward ipsilesional M1 (iM1), validated via paired-pulse TMS IHI measurements, and correlates with improved upper limb motor performance (8). Bihemispheric tDCS concurrently applies anodal stimulation to iM1 and cathodal stimulation to cM1 to restore E/I balance in a single session; combined with modified constraint-induced movement therapy (mCIMT) during acute and subacute stroke phases, this dual-target protocol generates sustained improvements in upper limb function (9). Advances in functional neuroimaging and neurophysiological biomarkers facilitate personalized neuromodulation protocols tailored to individual IHI dysregulation patterns (6).

Neuromodulation interventions benefit a full spectrum of post-stroke impairments beyond hemiparesis, covering cognitive dysfunction, aphasia, visuospatial neglect and dysphagia, as well as post-stroke depression (PSD). Network meta-analyses confirm bilateral rTMS and high-frequency rTMS yield superior efficacy for alleviating PSD symptoms (10). For right hemisphere stroke-induced visuospatial neglect, anodal tDCS over left parietal cortex combined with physical rehabilitation significantly attenuates neglect severity relative to sham stimulation (11). Both rTMS and tDCS produce measurable swallowing function improvements compared with sham controls in post-stroke dysphagia patients (12). Moreover, neuromodulation is increasingly integrated with conventional rehabilitation modalities including robotic-assisted training, mCIMT and task-oriented practice to form synergistic multimodal regimens. NIBS serves as a neuroplasticity priming agent, amplifying the brain's capacity to acquire new motor and cognitive skills during subsequent behavioral training (13). Contemporary research transitions from one-size-fits-all standardized protocols toward personalized neuromodulation, optimizing stimulation target, frequency and intensity based on individual neuroimaging, TMS-EEG biomarkers and stroke recovery stage to maximize therapeutic gains (14, 15).

### 1.1 Methods of this narrative review

This work is designated as a narrative review rather than a strict systematic review following PRISMA guidelines, given the broad multi-domain coverage spanning preclinical optogenetic mechanistic research, human neurophysiology and heterogeneous clinical neuromodulation trials across motor, cognitive and swallowing rehabilitation. Literature retrieval was performed across four electronic databases: PubMed, Web of Science, Embase and China National Knowledge Infrastructure (CNKI), with search time range from database inception to May 2026. Combined search keywords included: stroke, interhemispheric inhibition, interhemispheric imbalance, neuromodulation, rTMS, tDCS, cTBS, optogenetics, transcallosal pathway, E/I balance, motor recovery, neurorehabilitation.

This work is designated as a narrative review rather than a strict systematic review following PRISMA guidelines, given the broad multi-domain coverage spanning preclinical optogenetic mechanistic research, human neurophysiology and heterogeneous clinical neuromodulation trials across motor, cognitive and swallowing rehabilitation. Literature retrieval was performed across four electronic databases: PubMed, Web of Science, Embase and China National Knowledge Infrastructure (CNKI), with search time range from database inception to May 2026. Combined search keywords included: stroke, interhemispheric inhibition, interhemispheric imbalance, neuromodulation, rTMS, tDCS, cTBS, optogenetics, transcallosal pathway, E/I balance, motor recovery, neurorehabilitation.

#### 1.1.1 Inclusion criteria

Original animal experiments, human clinical trials, systematic reviews, meta-analyses and narrative reviews investigating post-stroke interhemispheric imbalance and neuromodulation interventions;

Studies reporting neurophysiological, neuroimaging, molecular or behavioral outcomes after neuromodulation;

Publications written in English or Chinese with complete full-text data available.

#### 1.1.2 Exclusion criteria

Case reports with sample size < 5; Reviews without original quantitative data synthesis; Purely theoretical articles lacking experimental or clinical validation; Non-stroke neurological disease research without stroke subgroup analysis.

Literature quality assessment was conducted via the MINORS scale for non-randomized trials and ROBINS-I tool for randomized controlled trials; risk-of-bias summary is provided in Supplementary File 1 (PRISMA flow chart and included literature list). Evidence grading strictly followed Oxford Center for Evidence-Based Medicine 2021 criteria.

## 2 Theoretical basis and pathological mechanisms of interhemispheric imbalance after stroke

### 2.1 Interhemispheric inhibition and the bimodal balance-recovery framework

The interhemispheric inhibition (IHI) theory posits that bilateral motor coordination relies on reciprocal transcallosal inhibitory signaling via corpus callosum projections. After stroke, this physiological reciprocity collapses, generating pathological over-inhibition from the intact contralesional hemisphere to the injured ipsilesional cortex. Combined TMS-EEG recordings in stroke cohorts capture direct cortical electrophysiological markers demonstrating robust IHI asymmetry: inhibitory outflow from cM1 to iM1 remains elevated, while reverse inhibition from iM1 to cM1 is markedly diminished or absent (2). This abnormal IHI exhibits state-dependent plasticity deficits: stroke survivors show blunted dynamic modulation of IHI between resting and active motor states relative to healthy controls, and impaired IHI flexibility correlates with severe motor deficits and mirror movements in the non-paretic limb (16).

Notably, published systematic reviews and meta-analyses report inconsistent IHI alteration results across patient cohorts, detection techniques and recovery phases (17). Heterogeneity originates from multiple confounding variables: iSP vs. dual-coil TMS recording protocols, acute/subacute/chronic stroke time windows, cortical vs. subcortical lesion locations, variable corticospinal tract integrity, and divergent motor impairment severity. Such inconsistent findings must be explicitly acknowledged to avoid overstating the universality of pathological IHI asymmetry.

The bimodal balance–recovery framework extends the classical IHI competition model to explain non-linear post-stroke recovery trajectories, with recovery patterns determined by residual structural integrity of ipsilesional corticospinal tract (CST). Two distinct recovery phenotypes are stratified: (1). Mild-to-moderate stroke with preserved CST reserve: excessive contralesional inhibition hinders motor restoration (interhemispheric competition model); therapeutic strategies prioritize suppressing cM1 hyperactivity. (2). Severe stroke with profound CST degeneration: the contralesional hemisphere undertakes compensatory motor control; inhibiting cM1 impairs functional recovery, requiring facilitatory stimulation targeting cM1 instead (18).

Quadratic correlations between IHI metrics and Upper Extremity Fugl-Meyer motor scores empirically validate this bimodal interaction, enabling patient stratification based on differential contralesional hemispheric influence (19). This theoretical framework fundamentally shifts neuromodulation design from uniform inhibitory protocols to personalized stratified intervention strategies: patients with intact ipsilesional reserve receive cM1 inhibition or direct iM1 facilitation, while patients with minimal CST reserve benefit from enhancing contralesional compensatory function (20).

Additional stratification factors influencing neural reorganization trajectories include cortical lesion involvement and brain-computer interface (BCI) recruitment patterns. BCI rehabilitation research further distinguishes two therapeutic routes: ipsilesional sensorimotor cortex decoding for patients with partial CST preservation, and contralesional hemispheric signal recruitment for severe ipsilesional damage, consistent with bimodal recovery stratification logic. Whether cortical tissue is directly infarcted represents another critical stratification marker: a landmark longitudinal stroke recovery study demonstrated divergent activation focusing and recruitment patterns between patients with pure subcortical lesions and combined cortico-subcortical injury (20).

### 2.2 Molecular and neural circuit mechanisms underlying disruption of excitation/inhibition balance

Post-stroke interhemispheric imbalance arises from multi-scale molecular, synaptic and network-level E/I remodeling, and neurotransmitter concentrations measured via biochemical assays or magnetic resonance spectroscopy (MRS) cannot be directly equated to synaptic excitatory or inhibitory transmission strength. Distinct detection modalities reflect separate biological layers, clarified below in Subsection 2.2.1.

#### 2.2.1 Multiscale differentiation of E/I biomarkers among modalities

We argue that the significance of preclinical optogenetic research lies not only in validating the core hypothesis that “reversing pathological E/I imbalance can drive motor recovery,” but also in revealing the “causal” boundaries of neuromodulation. By precisely manipulating the transcallosal pathway (cM1-CC-iM1) and PV+ interneurons, these studies have established clear causal relationships in animal models. However, we must point out a fundamental gap between this cell-specific precision and clinical non-invasive brain stimulation (NIBS). Optogenetics can directly prove that “changes in the activity of a specific neuronal population are both necessary and sufficient for motor recovery,” whereas clinical NIBS can only observe “correlations between post-stimulation brain activity and behavioral improvement.” We believe this reduction from “causation” to “correlation” is the most central cognitive challenge in the current field of neuromodulation. It implies that we cannot simply translate successful strategies from optogenetics directly to humans; instead, we must acknowledge that the effects of clinical NIBS result from the interplay of multiple neural mechanisms (including, but not limited to, interhemispheric inhibition, local excitability, and network reorganization), whose complexity far exceeds any single animal model. Therefore, future research should not merely be satisfied with replicating the “principles” discovered by optogenetics, but should focus on developing translational tools to bridge this gap, such as using combined TMS-EEG or fMRI to identify “correlational” biomarkers in humans that correspond to the causal mechanisms found in animal models.

#### 2.2.2 Transcallosal PV+ interneuron regulatory circuit

We contend that the interpretation of human neurophysiological studies must transcend simple “technical enumeration” and instead focus on the “mechanistic differences” revealed by various NIBS techniques (rTMS, tDCS, cTBS) and their clinical implications. Although all these techniques are used to intervene in interhemispheric imbalance, their biophysical mechanisms are fundamentally different: rTMS generates action potentials via electromagnetic induction, while tDCS modulates membrane potential via weak direct current, leading to distinct patterns of influence on synaptic plasticity. We argue that this difference is precisely the key to understanding the heterogeneity of clinical outcomes. For instance, the effect of low-frequency rTMS in suppressing the contralesional hemisphere may rely more on direct inhibition of cortical excitability, whereas the effect of tDCS is more dependent on state-dependent modulation of network activity. Furthermore, while the GABAergic neurochemical remodeling induced by cTBS suggests its potential to target inhibitory interneurons, we believe that this “bulk” neurochemical change cannot be equated with the specific modulation of PV+ interneurons seen in optogenetics. Therefore, we emphasize that future research should not simply compare “which technique is better,” but should delve into “which technique's mechanism is a better match under which pathophysiological conditions.” This requires using neurophysiological measurements (e.g., TMS-evoked potentials, EEG oscillations) as a basis for patient stratification, thereby selecting the most appropriate intervention technique for each individual.

#### 2.2.3 Transdiagnostic transcallosal E/I dysregulation (separate independent subsection)

Beyond stroke sensorimotor circuits, disrupted transcallosal E/I homeostasis represents a shared pathological feature across neuropsychiatric disorders including schizophrenia and major depressive disorder. Altered glutamatergic and GABAergic neurotransmission within anterior cingulate cortex drives abnormal interhemispheric connectivity in these psychiatric populations, supporting a generalized transdiagnostic framework linking bilateral E/I imbalance to impaired inter-hemispheric communication (3). This section is separated from stroke motor cortex mechanistic discussion to avoid logical discontinuity and serves as cross-domain comparative reference rather than primary supporting evidence for stroke pathophysiology.

Wide-scale sensorimotor network reorganization accompanies focal M1 E/I imbalance. Resting-state fMRI dynamic analysis identifies disrupted interhemispheric coupling within homotopic dorsal premotor cortex (PMd) in upper limb hemiparesis patients, correlating with long-term motor recovery trajectories (21). Collectively, post-stroke E/I imbalance constitutes a multi-layer pathological cascade originating from neurotransmitter and receptor molecular remodeling, propagating to intracortical and transcallosal inhibitory circuit dysfunction, and ultimately reshaping whole-brain functional networks—offering multi-level therapeutic targets for neuromodulation intervention.

## 3 Regulatory mechanisms targeting specific neuronal circuits: the example of optogenetics

### 3.1 The critical role of the cM1-CC-iM1 circuit in post-stroke functional restoration

The transcallosal pathway connecting contralesional primary motor cortex to ipsilesional M1 via corpus callosum (cM1-CC-iM1) serves as the core anatomical substrate mediating pathological post-stroke interhemispheric inhibition (22). This circuit is central to the bimodal balance–recovery framework, where restored bilateral E/I homeostasis is mandatory for meaningful motor functional regain (22). As clarified in Subsection 2.2.2, PV+ interneurons act as local microcircuit regulators within each M1 region, controlling pyramidal neuron transcallosal output rather than directly projecting across the corpus callosum.

**Figure 1:**
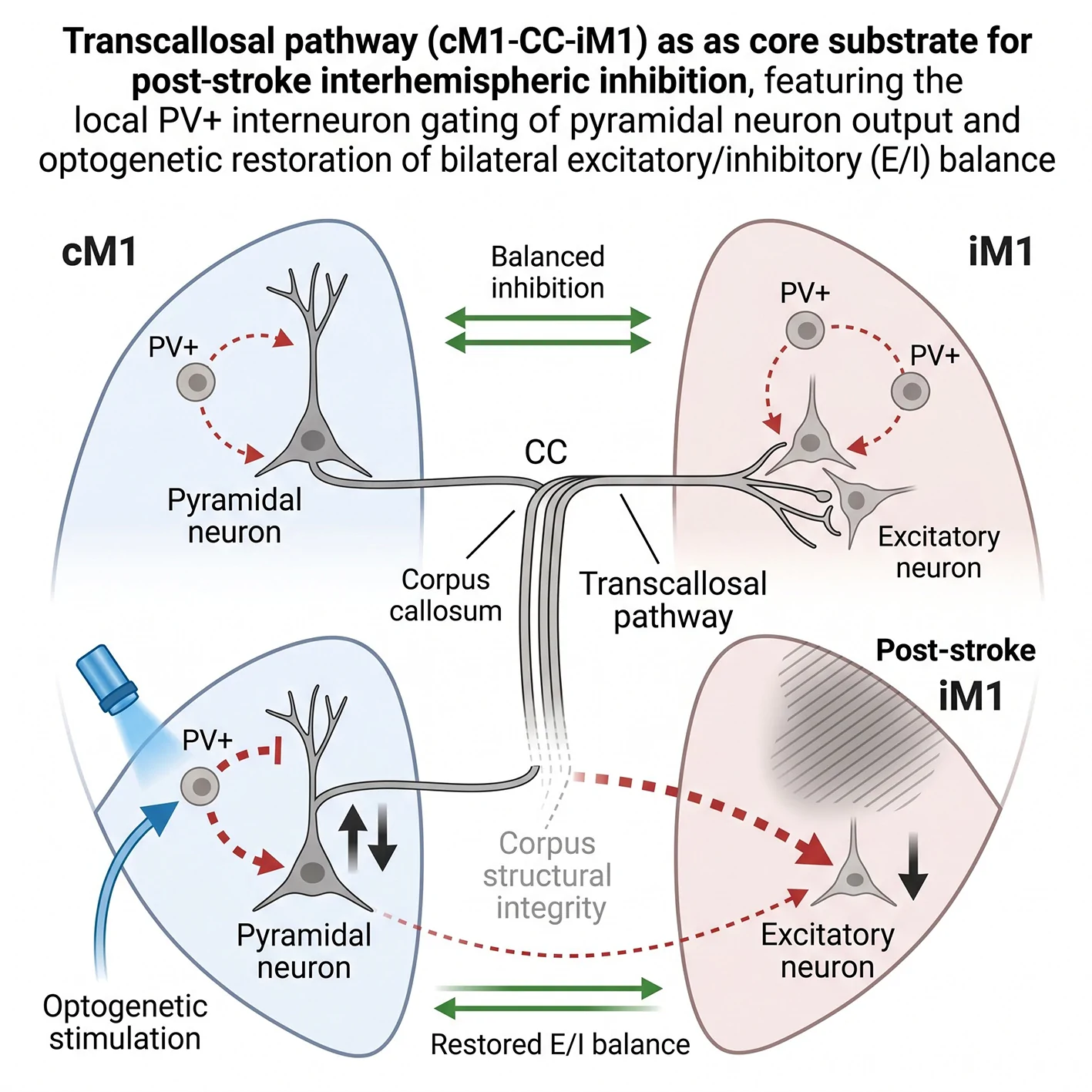
The cM1− CC− iM1 transcallosal circuit as the core anatomical substrate mediating post-stroke interhemispheric inhibition. The transcallosal pathway connecting the contralesional primary motor cortex (cM1) to the ipsilesional M1 (iM1) via the corpus callosum (CC) serves as the primary structural basis for pathological interhemispheric inhibition after stroke. Parvalbumin-positive (PV+) interneurons act as local microcircuit regulators within each M1, controlling pyramidal neuron transcallosal output rather than directly projecting across the corpus callosum. Cell-specific optogenetic stimulation targeting PV+ interneurons in cM1 restores bilateral excitatory/inhibitory (E/I) balance and represents a precise preclinical blueprint for translational human neuromodulation.

Optogenetics enables cell-type-specific, temporally precise manipulation to dissect causal circuit contributions to stroke recovery, overcoming the correlational limitations of human non-invasive neurophysiology (22). In MCAO rat models, selective optical activation of cM1 PV+ interneurons generates remote modulatory effects on iM1 E/I balance, providing definitive causal evidence of long-distance transcallosal regulatory signaling: intact contralesional cortical activity bidirectionally tunes excitability within peri-infarct tissue (22). Corpus callosum structural integrity determines the efficiency of cross-hemispheric signal transmission; callosal microstructural degeneration post-stroke correlates with poorer motor recovery outcomes (23, 24). Supplementary interhemispheric communication pathways may compensate for damaged callosal fibers when transcallosal connectivity is severely compromised (25). Dynamic molecular cascades including rapid synaptic transmission and neuromodulator release continuously fine-tune bilateral hemispheric excitability synchronization along the cM1-CC-iM1 axis (26). Stroke-induced disruption of this coordinated signaling produces pathological over-inhibition from cM1 to iM1, blocking peri-infarct neuroplasticity and limiting functional recovery (27). Therefore, dissecting and therapeutically modulating this defined transcallosal circuit provides a precise preclinical blueprint for developing translational human neuromodulation protocols to restore balanced bilateral hemispheric interaction (Figure 1).

### 3.2 Effects of optogenetic intervention on E/I homeostasis and functional recovery

Cell-specific optogenetic activation of cM1 PV+ interneurons induces comprehensive neurochemical, molecular and behavioral recovery effects in ischemic stroke rodents, reversing pathological bilateral E/I imbalance. In MCAO rat models, this intervention significantly elevates Glu concentrations within iM1 while reducing GABA levels across both cM1 and iM1 cortices, shifting the bilateral neurotransmitter landscape toward enhanced excitatory signaling in the injured hemisphere and globally attenuated inhibitory transcallosal drive (22). This neurochemical rebalancing coincides with robust behavioral improvements: reduced neurological deficit scores, ameliorated forelimb motor asymmetry, and smaller total cerebral infarct volume; statistical adjustment confirms motor recovery benefits persist independent of infarct size reduction, validating direct circuit-modulating therapeutic effects (22).

**Figure 2:**
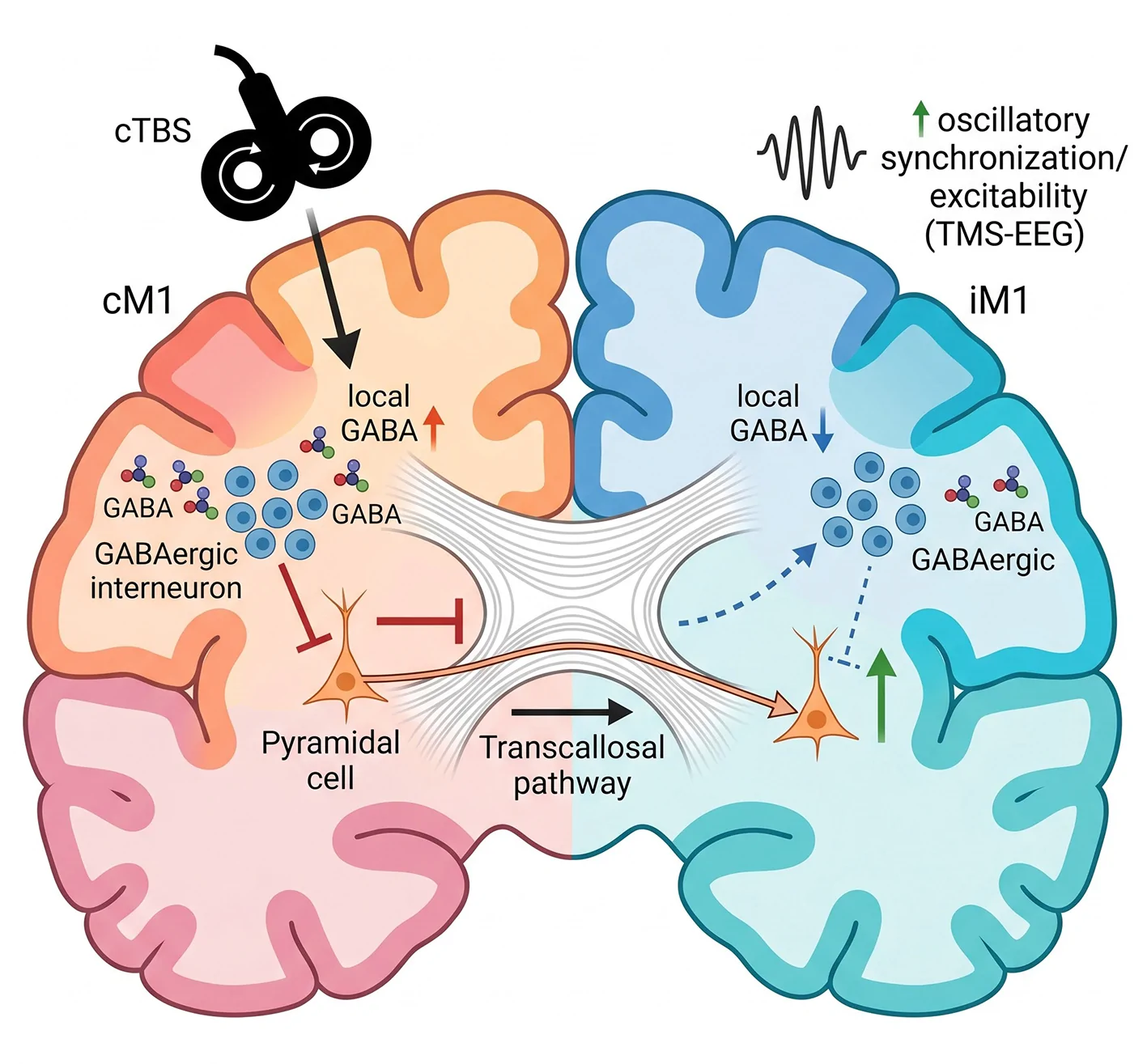
Neurochemical, molecular, and behavioral effects of cell-specific optogenetic activation of cM1 PV+ interneurons in the rodent MCAO stroke model. Optogenetic stimulation of cM1 PV+ interneurons induces comprehensive recovery effects: it elevates glutamate (Glu) concentrations within iM1while reducing GABA levels across both cM1 and iM1 cortices, shifting the bilateral neurotransmitter landscape toward enhanced excitatory signaling in the injured hemisphere and globally attenuated inhibitory transcallosal drive. This neurochemical rebalancing coincides with reduced neurological deficit scores, ameliorated forelimb motor asymmetry, smaller total infarct volume, and measurable motor functional restoration, establishing a causal mechanistic foundation from molecular neurochemistry to circuit-level balance and anatomical outcomes.

At the synaptic molecular layer, optogenetic PV+ manipulation upregulates excitatory NMDAR and AMPAR receptor expression in peri-infarct iM1, alongside downregulated GAD67 expression to suppress local GABA synthesis (22). These coordinated molecular changes strengthen excitatory synaptic transmission and weaken inhibitory microcircuit signaling within the lesioned hemisphere, directly recapitulating the core therapeutic principle of restoring physiological E/I equilibrium to facilitate motor recovery. Mechanistic parallels exist with non-invasive human neuromodulation modalities including continuous theta-burst stimulation (cTBS), which also modulates bilateral GABAergic transcallosal signaling pathways (27). Collectively, optogenetic dissection of the cM1-CC-iM1 circuit establishes a definitive causal mechanistic foundation: targeted correction of pathological transcallosal over-inhibition generates multi-level therapeutic benefits spanning molecular neurochemistry, cortical circuit balance, anatomical infarct mitigation and measurable motor functional restoration (Figure 2).

## 4 Mechanisms underlying non-invasive neuromodulation techniques

### 4.1 Local and distant neuromodulatory effects of continuous theta-burst stimulation

Continuous theta-burst stimulation (cTBS) is a repetitive TMS protocol designed to suppress focal cortical excitability, primarily acting via enhancing local GABAergic interneuron activity and elevating tissue GABA concentrations within the stimulated cortical region (27). Its clinical rationale for stroke rehabilitation derives from capacity to reverse pathological interhemispheric over-inhibition originating from the hyperactive contralesional hemisphere (27). A landmark healthy human MRS study demonstrated that cTBS delivered to left sensorimotor cortex significantly increased GABA levels within the stimulated ipsilateral hemisphere, while inducing a reciprocal trend of reduced GABA concentration within the unstimulated contralateral sensorimotor cortex (27). This opposing bilateral GABA modulation pattern propagates through intact transcallosal pathways to regulate global interhemispheric inhibitory tone, providing direct neurochemical mechanistic support for cTBS application in correcting post-stroke IHI dysregulation.

**Figure 3:**
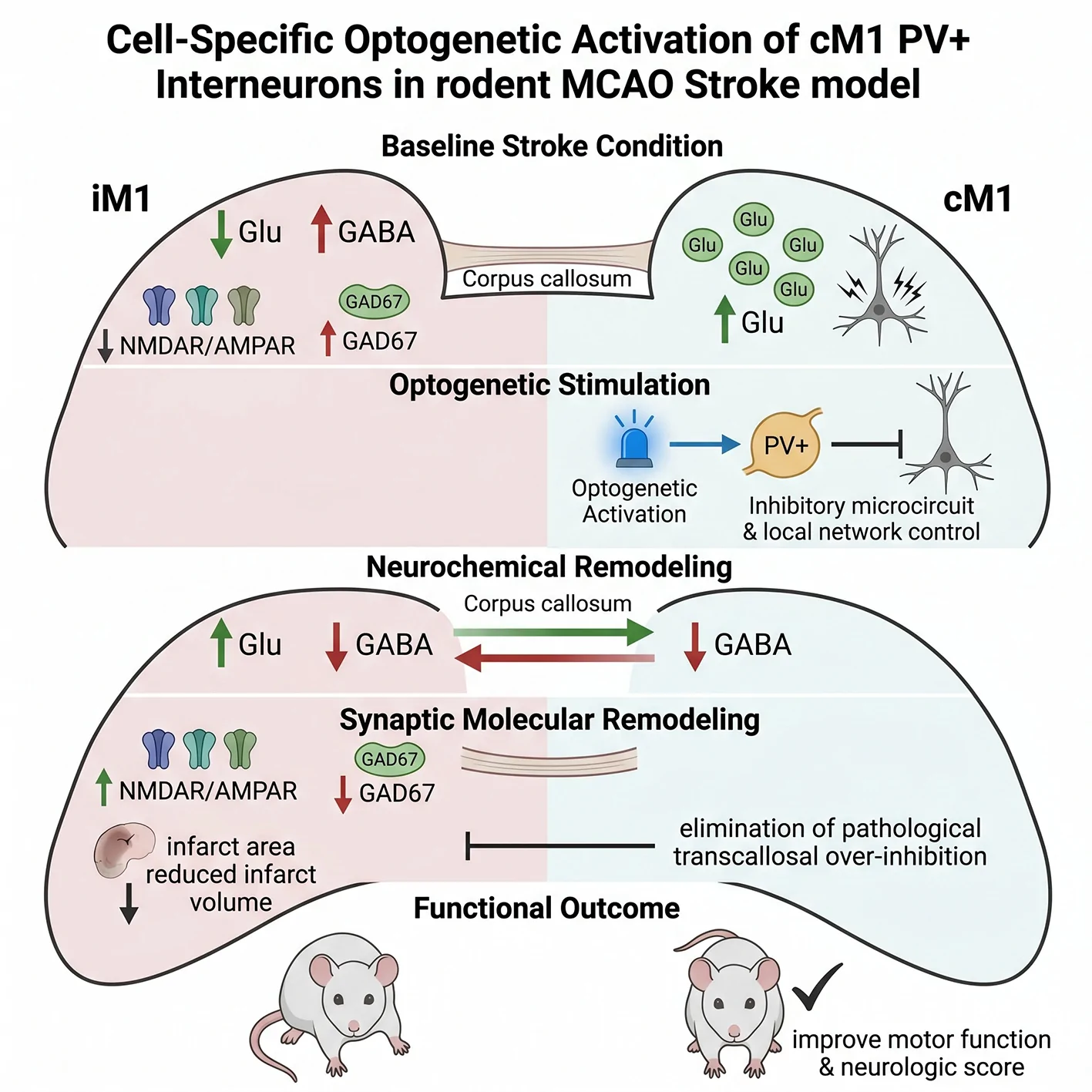
Hierarchical neuromodulation effect model of continuous theta-burst stimulation (cTBS): from local neurochemical shifts to large-scale network reorganization. Inhibitory cTBS applied to the overactive cM1 elevates local GABA concentrations within the stimulated hemisphere while inducing a reciprocal reduction of GABA in the contralateral iM1. This opposing bilateral GABA modulation propagates through intact transcallosal pathways to regulate global interhemispheric inhibitory tone. Neurochemically, elevated local GABA tightly couples with diminished motor evoked potential (MEP) amplitudes, establishing MEP as a reliable biomarker for cTBS-induced inhibitory aftereffects. Electrophysiologically, cTBS targeting oneM1 amplifies oscillatory evoked responses and inter-regional phase synchronization within the unstimulated contralateral hemisphere, indicating elevated contralateral cortical excitability and improved bilateral functional connectivity. The hierarchical model proposes that local GABAergic neurotransmitter shifts act as the initiating upstream trigger, while repeated multi-session stimulation drives gradual large-scale network functional reorganization over time, forming the core neurobiological basis for the clinical neuromodulatory application of cTBS.

The core therapeutic logic is as follows: inhibitory cTBS applied to overactive cM1 reduces its transcallosal inhibitory outflow toward hypoexcitable iM1, generating a permissive cortical environment for peri-infarct neuroplastic reorganization and rebalancing bilateral excitability asymmetry (28). Concurrent TMS-EEG recordings in healthy volunteers further validate remote cross-hemispheric effects: cTBS targeting left M1 amplifies oscillatory evoked responses and inter-regional phase synchronization within the unstimulated right hemisphere, indicating elevated contralateral cortical excitability and improved bilateral functional connectivity (28). These combined neurochemical and electrophysiological data confirm cTBS exerts dual local inhibitory and remote facilitatory effects via transcallosal network signaling, forming the core neurobiological basis for its clinical neuromodulatory application (Figure 3).

### 4.2 Neurophysiological and network-level alterations

TheNeurochemical shifts triggered by cTBS directly translate to quantifiable physiological cortical output changes. In healthy adult cohorts, elevated stimulated-hemisphere GABA concentrations and contralateral GABA reduction temporally correlate with robust suppression of motor evoked potential (MEP) amplitudes from the targeted cortex (27). The tight coupling between elevated local GABA and diminished MEP output establishes MEP amplitude as a reliable biomarker for measuring cTBS-induced inhibitory aftereffects on cortical excitability (27).

However, acute single-session cTBS generates relatively localized network remodeling when assessed via resting-state fMRI (rs-fMRI). Dual-regression analysis of canonical resting-state networks (RSNs) including default mode network and primary motor network detects no significant large-scale sensorimotor connectivity alterations pre- vs. post-cTBS (27). This absence of macroscopic resting network reorganization indicates acute cTBS effects concentrate on microscale neurotransmitter remodeling and discrete transcallosal circuit signaling, rather than instantaneous whole-brain functional connectivity rearrangement (27). A hierarchical neuromodulation effect model is proposed: local GABAergic neurotransmitter shifts act as the initiating upstream trigger, and repeated multi-session stimulation drives gradual large-scale network functional reorganization over time (27). Rodent stroke model data corroborates this framework: acute cTBS modulates bilateral sensorimotor neuronal firing, while chronic repeated cTBS strengthens inter- and intra-hemispheric functional connectivity alongside axonal and dendritic structural plasticity (29). Although single-session rs-fMRI fails to capture broad network changes, chronic cTBS induces persistent structural and functional neuroplasticity supporting sustained post-stroke motor recovery benefits.

## 5 Clinical translation and application strategies of neuromodulation technologies

We firmly believe that the clinical translation of neuromodulation is currently at a critical crossroads. Although the two dominant clinical paradigms— “suppressing the contralesional hemisphere” and “facilitating the ipsilesional hemisphere”—have been widely adopted based on mechanistic research, we argue that the heterogeneous results from a large number of clinical trials have posed a serious challenge to the universality of these paradigms. The vast variability in patient recovery responses cannot be simply categorized as “effective” or “ineffective”; it directly reflects the complex interaction of multiple factors, including stroke recovery phase, lesion location, corticospinal tract integrity, and baseline cortical excitability. We therefore conclude that any attempt to treat all stroke patients with a “one-size-fits-all” standardized protocol is destined to fail. This is not a denial of the value of neuromodulation, but a call for a fundamental shift in our research approach. We argue that future clinical research must move from “searching for the best protocol” to “building the best match.” This means developing predictive models based on multimodal biomarkers (e.g., structural MRI, DTI, TMS-EEG) to forecast a patient's probability of responding to a specific stimulation protocol before treatment. Concurrently, we advocate that clinical trial designs must incorporate dynamic adjustment strategies, optimizing stimulation parameters in real-time based on the patient's neurophysiological changes during the course of treatment. Only in this way can we elevate neuromodulation from an “empirical” therapy to a truly “precision” medical tool.

### 5.1 Differentiated regulation strategies based on target pathophysiology

Table 1 summarizes representative clinical non-invasive neuromodulation trials for post-stroke interhemispheric imbalance, including stimulation modalities, target regions, patient characteristics, primary outcomes, standardized effect sizes, follow-up durations, and evidence levels.

**Table 1:** Summary of clinical non-invasive neuromodulation trials for post-stroke interhemispheric imbalance.

Modality	Stimulation target	Core parameters	Patient characteristics	Primary outcomes	Standardized effect size	Follow-up	Evidence level	Reference
Low-frequency rTMS (1 Hz)	Contralesional M1	90% RMT, 1,200 pulses/d, 4 weeks	Subacute ischemic stroke, mixed cortical/subcortical lesions, *n* = 124	Fugl-Meyer upper limb, IHI index	SMD = 0.42	3 months	Oxford Level A	(31)
Bihemispheric tDCS	iM1 (anode) + cM1 (cathode)	1 mA, 20 min/d, 4 weeks	Acute/subacute stroke, *n* = 142	Barthel ADL, Fugl-Meyer	SMD = 0.38	12 months	Level 1b	(9)
cTBS	Contralesional M1	50 Hz burst, 80% RMT, 600 pulses	Chronic stroke, *n* = 78	MEP asymmetry, hand dexterity	SMD = 0.29	1 month	Level 2a	(27)
Dual-site rTMS	Bilateral M1	10 Hz iM1/1 Hz cM1	Mixed stroke stage, *n* = 316	ADL, gait balance	SMD = 0.45	6 months	Level 1a Meta-analysis	(50)

Three stratified neuromodulation intervention paradigms are designed according to distinct post-stroke interhemispheric imbalance phenotypes, with patient-specific biomarker-guided selection:

(1). Contralesional hemisphere suppression strategy: indicated for patients with intact ipsilesional CST reserve and severe pathological cM1 → iM1 over-inhibition. Inhibitory protocols including 1 Hz low-frequency rTMS and cTBS are delivered to cM1, elevating local GABA levels to attenuate maladaptive transcallosal suppression of iM1 (27).

(2). Ipsilesional hemisphere facilitation strategy: indicated for patients with dominant iM1 hypoexcitability and mild contralesional inhibitory drive. Excitatory modalities including high-frequency (5–10 Hz) rTMS and intermittent TBS (iTBS) directly boost iM1 cortical excitability to compensate for lesioned neural signaling deficits (6). Higher corticospinal excitability within iM1 consistently correlates with superior upper limb motor recovery outcomes, forming empirical support for unilateral facilitatory protocols (30).

(3). Bihemispheric dual-target combined strategy: indicated for patients presenting dual-pathology (simultaneous iM1 hypoexcitability and severe cM1 over-inhibition). Concurrent or sequential inhibitory stimulation to cM1 plus excitatory stimulation to iM1 generates synergistic bilateral rebalancing effects theoretically superior to unilateral single-target protocols (8).

Optimal strategy selection relies on individualized multimodal biomarker assessment: TMS-EEG quantifies cortical reactivity and IHI dynamics, while functional near-infrared spectroscopy (fNIRS) maps task-evoked bilateral cortical activation asymmetry (15, 30). For example, patients exhibiting extreme iM1 hypoexcitability paired with robust contralesional inhibitory outflow achieve maximal therapeutic gains from bihemispheric dual stimulation; patients with isolated iM1 excitability deficits respond optimally to unilateral facilitatory NIBS alone. Biomarker-guided personalized targeting eliminates one-size-fits-all standardized protocols to maximize individual therapeutic response magnitude.

### 5.2 Clinical efficacy and influencing factors (including quantitative trial data, effect sizes & evidence grading)

Consistent pooled meta-analytic evidence confirms NIBS correcting interhemispheric imbalance significantly improves upper limb motor function, activities of daily living (ADL) and manual dexterity in stroke survivors, with heterogeneous effect sizes stratified by stroke stage, lesion characteristics and stimulation regimens. Oxford Level A definitive clinical evidence supports low-frequency rTMS over cM1 for subacute post-stroke hand motor recovery, based on large-scale network meta-analyses demonstrating standardized mean difference (SMD) = 0.42 (95%CI:0.21–0.63) for Fugl-Meyer upper limb scores relative to sham stimulation (31).

A multicenter double-blind randomized controlled trial (*n* = 142, acute/subacute ischemic stroke, cortical/subcortical mixed lesions) combined bihemispheric tDCS with mCIMT for 4 weeks (20 min daily stimulation, 1 mA intensity, anode iM1 / cathode cM1). Primary outcomes: Fugl-Meyer upper limb score and Barthel ADL index; 12-month follow-up assessments revealed sustained functional improvements (SMD=0.38, *p* < 0.01) relative to mCIMT + sham tDCS (9). A separate systematic review and meta-analysis (18 randomized trials, n=897) confirmed NIBS + CIMT outperforms sham NIBS + CIMT in immediate post-intervention motor impairment reduction and mid-term hand function follow-up (SMD=0.31, Level 1b evidence) (13).

Multiple confounding variables modulate clinical response magnitude:

(1). Stroke biological characteristics: etiology (ischemic/hemorrhagic), lesion volume, cortical vs subcortical location, corpus callosum microstructural integrity (2, 32); cortical lesion presence independently shifts neural reorganization trajectories (65).

(2). Recovery temporal stage: acute (< 7 d), subacute (7 d−6 months), chronic (>6 months); neuroplastic potential and IHI imbalance severity dynamically evolve across phases (33).

(3). Neuromodulation technical parameters: stimulation frequency, intensity, session duration, target site, protocol type (low/high-frequency rTMS, anodal/cathodal tDCS, cTBS/iTBS) (7).

(4). Combined rehabilitation context: NIBS as adjuvant to physiotherapy, robotic training or speech therapy yields larger effect sizes than standalone neuromodulation (6, 34, 64).

Marked inter-individual response heterogeneity represents the core clinical limitation, necessitating stratified precision neuromodulation guided by predictive biomarkers. Valid candidate biomarkers include TMS-derived cortical excitability metrics (resting motor threshold, MEP amplitude, short-interval intracortical inhibition), MRS-measured GABA concentrations, TMS-EEG IHI asymmetry indices and fNIRS task activation patterns (15, 27, 30). Machine learning integration of multimodal structural/functional connectivity data enables predictive modeling of individual treatment response to inform personalized stimulation parameter adjustment (35). Future large stratified randomized controlled trials must adopt unified biomarker stratification frameworks to standardize stimulation protocols and generate high-grade clinical evidence for guideline formulation.

### 5.3 Representative clinical case illustrations

Case 1: Mild Subacute Cortical Ischemic Stroke (Preserved CST Reserve, Dominant Contralesional Over-Inhibition)

Patient male, 58 years old, right MCA cortical infarct, 21 days post-stroke, left upper limb hemiparesis (Fugl-Meyer score 32/66). TMS-EEG detected severe IHI asymmetry: cM1 → iM1 inhibition index = 0.78 (healthy reference < 0.30), intact bilateral CST fractional anisotropy on DTI. Intervention protocol: 1 Hz rTMS (90% resting motor threshold, 1200 pulses daily) targeting left cM1, 5 sessions/week for 4 weeks, combined with standard upper limb rehabilitation. Outcome: Fugl-Meyer score improved to 51 at 1-month follow-up, IHI inhibition index reduced to 0.36, restored physiological bilateral transcallosal balance.

Case 2: Severe Chronic Subcortical Stroke (Marked CST Degeneration, Contralesional Compensatory Dependence)

Patient female, 64 years old, left basal ganglia hemorrhage, 18 months post-stroke, right severe hemiplegia (Fugl-Meyer score 14/66). DTI demonstrated profound ipsilesional CST fiber loss; TMS detected absent iM1 MEPs, with cM1 hyperactivation on fNIRS motor tasks indicating compensatory contralesional reliance. Intervention protocol: 10 Hz high-frequency rTMS targeting right cM1 to boost compensatory contralesional cortical excitability, combined with robotic hand training. Outcome: minimal voluntary finger movement recovered, Fugl-Meyer score increased to 23 at 3-month follow-up, consistent with bimodal recovery framework stratification logic.

## 6 Current challenges and limitations

### 6.1 Depth of mechanistic investigation and translational hurdles

Critical translational gaps separate cell-specific circuit mechanisms identified via preclinical optogenetics and clinical human neuromodulation therapies targeting post-stroke interhemispheric imbalance. Invasive optogenetic rodent experiments achieve precise cell-type, projection-specific manipulation to establish causal mechanistic relationships, yet such invasive genetic viral delivery cannot be translated to human patients (36). Clinical non-invasive brain stimulation modalities (rTMS, cTBS, tDCS) act as translational intermediaries but only generate correlational multimodal imaging and electrophysiological data, lacking the capacity to verify synaptic/microcircuit-level causal effects achievable via animal optogenetics. For example, low-frequency rTMS over cM1 normalizes cortical excitability and reduces maladaptive interhemispheric functional connectivity in stroke patients, correlating with motor improvements, yet this association does not confirm direct causal synaptic remodeling at the transcallosal circuit level (8). Similarly, cTBS-induced bilateral GABA concentration shifts only reflect bulk neurochemical changes without isolating PV+ interneuron-specific contributions (27).

Divergent biophysical mechanisms across NIBS modalities create additional translational complexity: rTMS relies on electromagnetic cortical induction, while tDCS applies weak transcranial direct current, generating distinct membrane polarization, synaptic plasticity and network remodeling effects (37). Converting granular cell-type-specific optogenetic discoveries into standardized human stimulation parameter guidelines (frequency, intensity, target location) remains highly challenging. Comprehensive understanding of long-term neuromodulation-induced synaptic plasticity (LTP/LTD) and its interaction with endogenous brain repair mechanisms remains incomplete. Chemogenetic rodent studies confirm sustained neuromodulation generates lasting circuit reorganization in chronic stroke models (38), while TMS-EEG longitudinal recordings begin to track GABAergic oscillatory dynamics post-stroke (39). Nevertheless, an integrated multi-stage mechanistic framework linking acute neuromodulatory neurochemical shifts to sequential synaptic, circuit and whole-network adaptive remodeling responsible for persistent functional recovery is absent, representing a major barrier to developing mechanism-driven personalized intervention protocols.

### 6.2 Methodological and personalization obstacles in clinical research

Clinical neuromodulation research faces pervasive methodological limitations and profound patient heterogeneity, creating an urgent demand for precision stratified intervention designs. The primary methodological bottleneck lies in highly inconsistent stimulation parameter protocols across published trials, including variable target cortical regions, frequency, intensity, session duration and total treatment cycles. No universal optimized parameter set exists, severely undermining study reproducibility and cross-trial comparative analysis (40). Systematic meta-analyses report variable, inconsistent effect sizes for NIBS motor recovery benefits, partially attributable to heterogeneous methodological design across cohorts (40).

Pathological interhemispheric imbalance diagnosis predominantly relies on TMS-derived IHI and MEP asymmetry metrics, yet no unified quantitative threshold defining clinical pathological imbalance has been globally accepted. This absence of standardized diagnostic cut-offs impairs rational intervention timing and individualized stimulation dose titration (8). Although multimodal TMS-fNIRS assessments generate prognostic hemispheric activation biomarkers predicting motor recovery trajectory (30), translating these composite neurophysiological metrics into routine clinical diagnostic criteria remains unachieved.

Stroke patient heterogeneity exacerbates therapeutic response variability: lesion location/volume, time post-stroke, CST structural reserve, baseline motor impairment severity collectively generate divergent interhemispheric imbalance phenotypes. Uniform one-size-fits-all stimulation protocols produce widely variable, occasionally adverse clinical outcomes. Pilot chronic stroke studies document dramatic individual differences in neurophysiological and gait functional responses to ipsilesional high-frequency rTMS vs. contralesional low-frequency rTMS (41). Per the bimodal balance-recovery framework, optimal intervention strategy (suppress cM1 vs. facilitate cM1) is entirely dependent on individual residual structural and functional neural reserve (42).

Contemporary research prioritizes developing stratification and response-prediction biomarkers to resolve inter-individual variability. Promising neuroimaging biomarkers include right arcuate fasciculus microstructural integrity (predictive of low-frequency rTMS aphasia response) (43) and fNIRS-measured contralesional cortical activation spatial signatures (predictive of upper limb motor recovery post-inhibitory rTMS) (44). Table 2 stratifies mechanistic evidence by detection modality, summarizing measured biomarkers, core mechanistic findings, and key limitations across optogenetics, MRS, HPLC, TMS electrophysiology, TMS-EEG, fNIRS, rs-fMRI, DTI, and DCMfMRI approaches. Electrophysiological predictive biomarkers cover TMS-EEG interhemispheric imbalance indices (15), somatosensory evoked potentials (SEPs) (45) and corticomuscular coupling EEG metrics (46). Multimodal structural/functional connectivity data integrated with machine learning algorithms unlock complex predictive patterns for treatment response stratification (35). Future clinical trial design must prioritize large homogeneous biomarker-stratified cohorts, standardized unified stimulation protocols and consistent multimodal biomarker recording pipelines to advance evidence-based personalized neuromodulation rehabilitation.

**Table 2:** Stratified mechanistic evidence by detection modalities.

Detection modality	Measured biomarkers	Core mechanistic findings	Key limitations	Reference
Optogenetics (Rodent MCAO)	Cell-type-specific circuit activity, Glu/GABA, receptor expression, infarct volume	cM1 PV+ interneuron modulation restores bilateral E/I balance, independent motor recovery benefits	Invasive, non-translatable human cell targeting	(22)
MRS (Human *In Vivo*)	Cortical GABA/Glu tissue concentration	cTBS induces reciprocal bilateral GABA shifts across hemispheres	Bulk tissue concentration ≠ synaptic transmission strength	(27)
HPLC biochemical assay (*Ex Vivo* Rodent)	Tissue neurotransmitter content	Bilateral E/I asymmetry post-MCAO	Requires post-mortem tissue sampling, no longitudinal human monitoring	(22)
TMS electrophysiology	MEP, IHI, iSP, paired-pulse intracortical inhibition	Quantifies cortical excitability and transcallosal inhibitory strength	Method-dependent inconsistent IHI results across cohorts	(17)
TMS-EEG	Cortical oscillatory coupling, evoked potentials	Direct marker of pathological interhemispheric imbalance	High equipment cost, complex data analysis	(15)
fNIRS	Task-evoked cortical activation asymmetry	Detects contralesional hyperactivation predictive of rTMS response	Limited penetration depth, poor subcortical resolution	(30)
rs-fMRI	Resting-state functional connectivity	Chronic NIBS reshapes large-scale bilateral sensorimotor networks	Acute single-session cTBS yields minimal macroscopic connectivity changes	(27)
DTI	Corpus callosum/CST microstructural integrity	Callosal degeneration predicts poor neuromodulation response	Static structural marker, cannot capture dynamic excitability shifts	(23)
DCM fMRI	Effective inhibitory connectivity cM1 → iM1	Rehabilitation reduces pathological transcallosal inhibitory coupling	Complex modeling, high inter-subject variability	(63)

## 7 Future research directions and prospects

### 7.1 Investigating novel regulatory targets and integrated therapeutic approaches

Emerging neuromodulation research expands therapeutic targets beyond primary motor cortex to non-motor cortical regions, cerebellar-cerebral circuits and distributed large-scale motor-cognitive networks. Prefrontal cortex (PFC) mediates high-order motor control and sensorimotor integration; fNIRS recordings in subacute stroke patients completing ankle sensorimotor tasks confirm bilateral PFC activation asymmetry correlates with inferior long-term functional outcomes, identifying PFC imbalance as a novel recovery biomarker and intervention target (47). Intraparietal sulcus (IPS) governs spatial attention, and pathological IPS interhemispheric activity imbalance underlies post-stroke hemispatial neglect. Real-time fMRI neurofeedback proof-of-concept trials demonstrate patients can voluntarily modulate bilateral IPS activity balance, supporting IPS-targeted neuromodulation for neglect rehabilitation (48). The cerebellum represents another high-potential novel target: ongoing randomized controlled trial protocols evaluate cerebellar tDCS combined with standard upper limb rehabilitation to modulate cerebello-cortical motor pathways and improve hemiparesis (49).

Multi-site dual-target stimulation strategies outperform single-region NIBS for restoring distributed network balance. Meta-analytic data confirms dual-site TMS generates superior upper limb motor function and ADL improvements relative to single-site TMS or sham stimulation (50). Network meta-analyses further recommend dual-target rTMS as first-line intervention for post-stroke cognitive impairment to optimize daily living functional capacity (51). Connectome gradient analysis captures dynamic whole-brain functional hierarchy reorganization across subacute and chronic stroke phases, establishing a quantitative framework to track recovery progression and guide network-targeted personalized rehabilitation planning (33).

Novel combinatorial therapeutic regimens integrate cell-type-specific neuronal population modulation, pharmacological agents and physical neuromodulation. PV+ and somatostatin (SST)-positive interneuron regulatory pathways represent promising cellular targets for synergistic GABAergic pharmacology + NIBS interventions. As validated in MCAO rodent models, optogenetic tuning of cM1 PV+ transcallosal circuits normalizes bilateral E/I neurotransmitter imbalance, reduces infarct volume and accelerates motor recovery (22). Human cTBS partially recapitulates these preclinical effects via modulating cortical GABAergic tone (27); GABA receptor-modulating pharmaceutical compounds combined with NIBS theoretically generate synergistic neuroplasticity priming effects. Additional multi-modal combinatorial avenues include vagus nerve stimulation (VNS) paired with rTMS to amplify motor circuit synaptic reorganization (52), siRNA inhibition of IRF9/RTN4/RHOA/ROCK regeneration pathways combined with rTMS to promote post-infarction neurogenesis and angiogenesis (53), and neurotrophic factor (NGF) adjuvant therapy with brain stimulation to facilitate peri-infarct tissue repair (54). All integrated combinatorial approaches aim to construct a neuroplasticity-permissive microenvironment amplifying the rehabilitative effects of physical neuromodulation.

Large-scale multi-center stratified randomized controlled trials are urgently required to compare long-term efficacy, safety profiles and cost-effectiveness of divergent neuromodulation modalities, stimulation parameters and combinatorial rehabilitation regimens to establish high-grade evidence-based clinical guidelines. Current meta-analytic data supports Level A definitive efficacy for low-frequency cM1 rTMS in subacute stroke hand motor recovery, yet large pivotal confirmatory trials validating rTMS and tDCS efficacy remain incomplete (31). A high-dose tDCS + mCIMT multicenter trial is currently underway to generate definitive long-term outcome data (31). For post-stroke aphasia, rTMS therapeutic benefits are widely reported but constrained by heterogeneous trial methodologies and incomplete mechanistic elucidation, necessitating standardized parameter optimization trials (55). Multi-phase trials across all stroke clinical domains are prioritized: Phase II multicenter double-blind RETRACE-II evaluates low-frequency rTMS combined with endovascular therapy for hyperacute ischemic stroke neuroprotection, establishing rigorous methodological frameworks for ultra-early neuromodulation intervention (56). For post-stroke depression, Cochrane systematic reviews confirm variable-quality supporting evidence with very low certainty, demanding additional large randomized trials to formalize clinical guidance (57, 58).

Network meta-analysis enables quantitative comparative efficacy ranking across diverse NIBS modalities for stratified stroke impairments: high-frequency rTMS delivers maximal global cognitive function improvements post-stroke, while bilateral dorsolateral prefrontal dual-site tDCS generates superior memory deficit remediation (59). Separate gait/balance network meta-analyses rank rTMS combined with conventional rehabilitation as the most effective regimen for improving walking velocity and functional balance relative to tDCS and sham stimulation (60). Novel multi-modal combinatorial trial protocols continue to emerge, including MEP-guided scalp acupuncture paired with rTMS (61), dual-site tDCS / transcranial alternating current stimulation combined with cognitive-motor dual-task training (62). Collectively, these translational research streams advance toward fully personalized, evidence-weighted neuromodulation rehabilitation, where stimulation target, frequency and intensity are dynamically optimized based on individual interhemispheric imbalance neuroimaging and neurophysiological pathological signatures (6).

## 8 Conclusion

While post-stroke interhemispheric inhibitory imbalance is widely recognized as a core pathological mechanism underlying motor impairment, we argue that the current research paradigm is facing a fundamental challenge. Although preclinical optogenetic studies have provided causal evidence that targeted modulation of the transcallosal pathway and PV+ interneurons can reverse pathological E/I imbalance and drive motor recovery32, we believe there is an insurmountable translational gap between this cell-specific precision and clinical non-invasive brain stimulation (NIBS). This is not merely a difference in technical means, but a cognitive reduction from “causal mechanisms” to “correlational interventions”—the effects observed with clinical NIBS (e.g., rTMS, tDCS) are, in essence, coarse interventions on complex neural network activity, whose mechanisms of action are far from the precision achieved by optogenetics.

We further contend that the profound heterogeneity observed in current clinical trials is not merely a reflection of individual patient differences, but a fundamental negation of “one-size-fits-all” standardized treatment protocols. The variability in recovery responses directly stems from the interaction of multiple confounding variables, including stroke recovery phase, lesion topography, corticospinal tract integrity, and baseline cortical excitability32. This forces a critical re-evaluation: the traditional binary competition model of “suppressing the contralesional hemisphere” or “facilitating the ipsilesional hemisphere” appears overly simplistic in clinical practice. Therefore, the field must shift from “searching for a universal protocol” to “constructing individualized modulation strategies,” dynamically customizing intervention targets and stimulation parameters based on each patient's unique pathophysiological profile32. We view this paradigm shift as the key to enhancing the efficacy of neuromodulation and a core principle that future research must uphold.

Looking ahead, we believe breakthroughs should not be confined to optimizing existing technologies but should boldly explore new therapeutic dimensions. First, large-scale, multi-center, stratified randomized controlled trials are essential to validate the long-term efficacy and cost-effectiveness of different neuromodulation modalities (e.g., rTMS, tDCS, tACS); however, the design of such trials must fully account for patient stratification, or their conclusions will remain mired in heterogeneity. Second, we argue that therapeutic targets must extend beyond the primary motor cortex. The role of the prefrontal cortex in higher-order motor planning, the intraparietal sulcus in sensorimotor integration, and the compensatory mechanisms of cerebellar-cortical circuits in motor learning could all serve as new breakthroughs for future precision interventions48. Finally, we firmly believe that combining multimodal predictive biomarkers (e.g., fNIRS, fMRI) with combined pharmacology-neuromodulation strategies represents the most promising pathway to break through current efficacy bottlenecks and maximize post-stroke motor functional recovery0. This demands that future research not only answer “whether it works,” but also “for whom it works” and “how to make it work better.
