# Supplementary material for: Research progress on the mechanisms and clinical applications of neuromodulation techniques for intervening in interhemispheric imbalance after stroke
Source: Front Neurol. 2026 Sep 7;17:1890806. doi: 10.3389/fneur.2026.1890806 (PMC13591252; doi:10.3389/fneur.2026.1890806)

Supplementary Material

1 Supplementary Data

Supplementary Material should be uploaded separately on submission. Please include any supplementary data, figures and/or tables.

Supplementary material is not typeset so please ensure that all information is clearly presented, the appropriate caption is included in the file and not in the manuscript, and that the style conforms to the rest of the article.

2 Supplementary Figures and Tables

For more information on Supplementary Material and for details on the different file types accepted, please see here.

2.1 Supplementary Figures

Supplementary Figure 1. The transcallosal pathway connecting contralesional primary motor cortex to ipsilesional M1 via corpus callosum (cM1-CC-iM1) serves as the core anatomical substrate mediating pathological post-stroke interhemispheric inhibition. Critical clarification of PV+ interneuron localization and projection patterns: PV+ inhibitory interneurons reside within local microcircuits of cM1 and iM1, rather than forming direct transcallosal axonal projections through the corpus callosum. These local PV+ neurons modulate the firing of pyramidal tract projection neurons whose long-range axons cross the corpus callosum to form the cM1-CC-iM1 transcallosal pathway. In intact healthy brain, balanced reciprocal inhibition is maintained; after focal ischemic stroke in iM1, pyramidal output from cM1 becomes overactive, delivering excessive GABAergic inhibitory signals across corpus callosum to suppress residual excitatory neurons in peri-infarct iM1, forming pathological interhemispheric imbalance. Corpus callosum structural integrity determines the efficiency of cross-hemispheric signal transmission; callosal microstructural degeneration post-stroke correlates with poorer motor recovery outcomes. Optogenetic targeting of cM1-local PV+ interneurons can normalize overactive pyramidal projection firing, relieve pathological transcallosal suppression and restore bilateral E/I equilibrium.

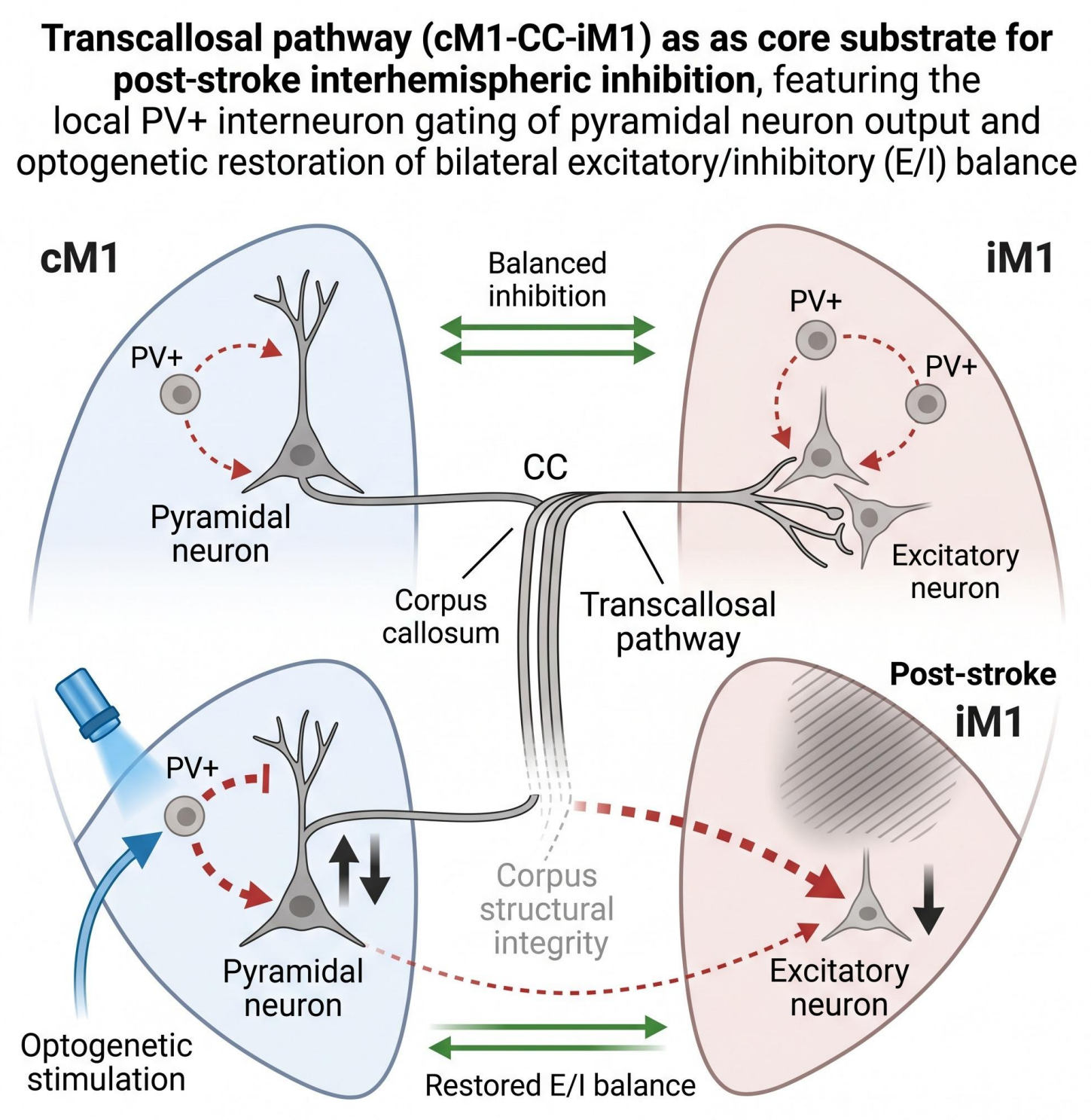

Supplementary Figure 2. Pre-stroke baseline neurotransmitter & receptor state; 2. Post-stroke pathological E/I asymmetry; 3. Optogenetic intervention-induced molecular correction; Markers: Glu = glutamate (excitatory); GABA = γ-aminobutyric acid (inhibitory); NMDAR/AMPAR = excitatory ionotropic receptors; GAD67 = rate-limiting GABA synthetase

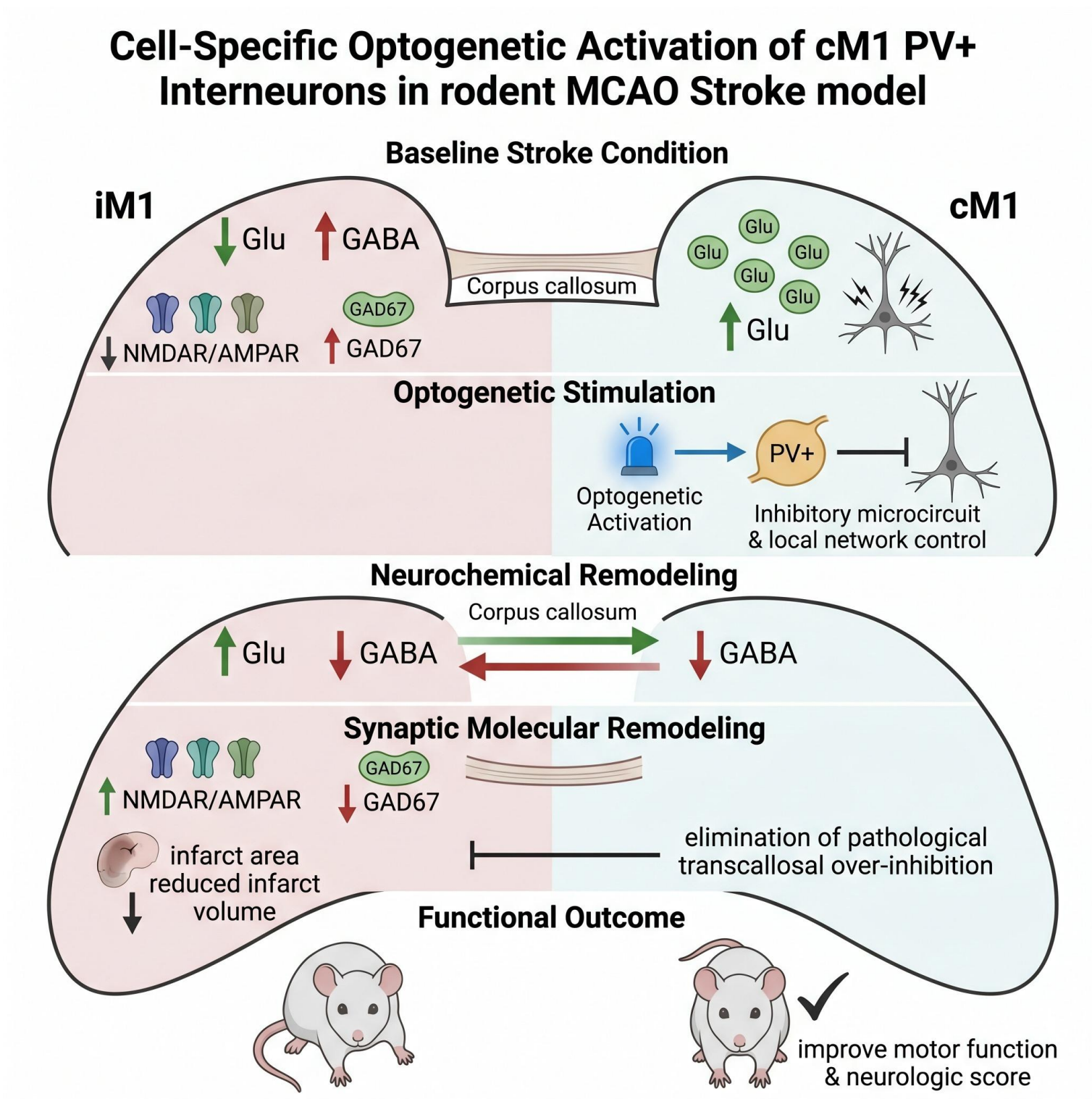

Supplementary Figure 3. Stimulation coil for cTBS placed over contralesional M1; 2. Stimulated hemisphere (cM1): increased GABA concentration; 3. Unstimulated ipsilesional hemisphere (iM1): decreased GABA concentration; 4. Transcallosal inhibitory fiber bundle; Color gradient: high GABA (dark red) /low GABA (light blue); Correction arrow: normalized interhemispheric inhibitory signal after cTBS

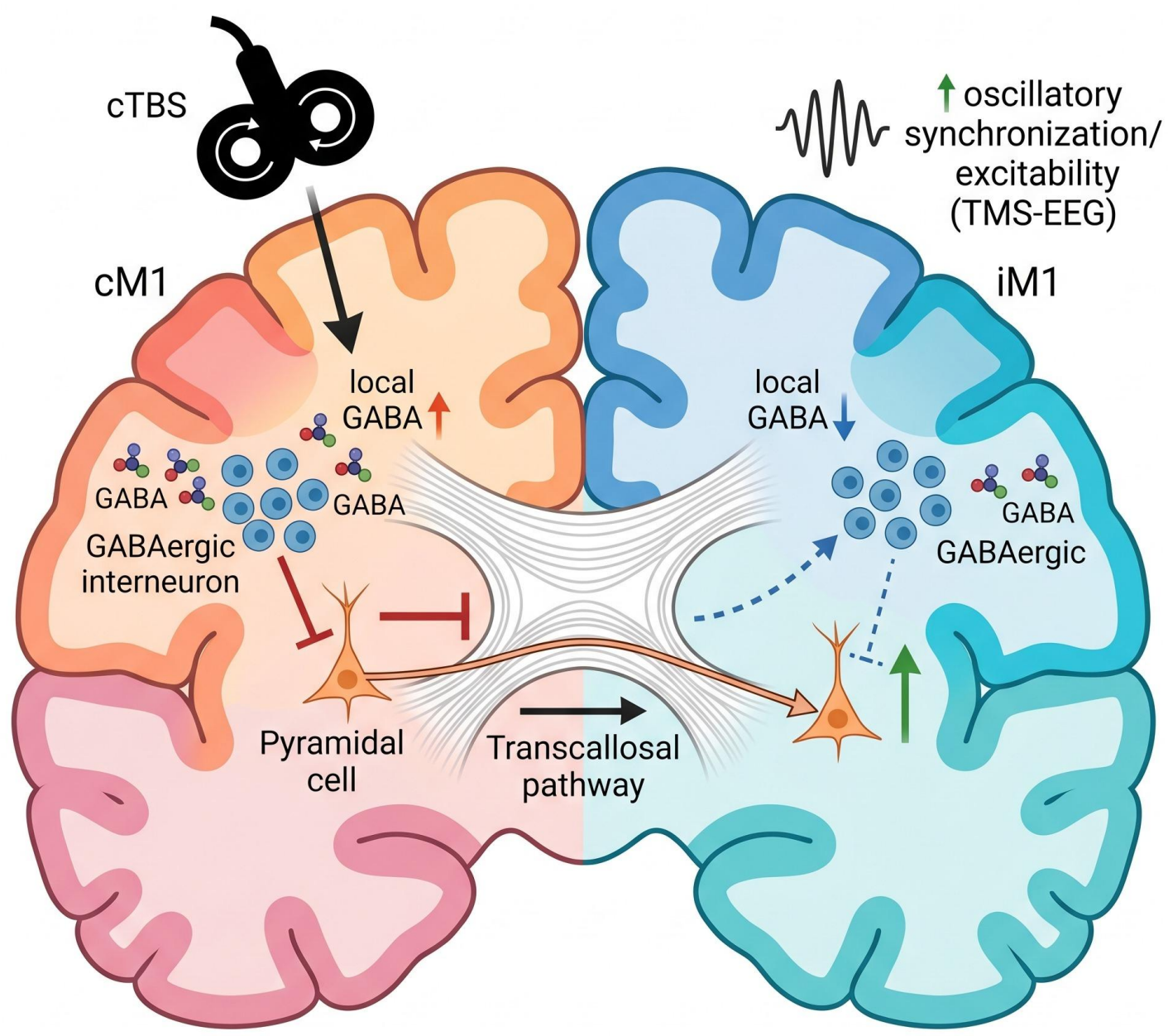

Supplement: Supplementary file 1 [file Data_Sheet_1.pdf]
